# Maternal Resveratrol Supplementation Prevents Cognitive Decline in Senescent Mice Offspring

**DOI:** 10.3390/ijms20051134

**Published:** 2019-03-06

**Authors:** Vanesa Izquierdo, Verónica Palomera-Ávalos, Sergio López-Ruiz, Anna-Maria Canudas, Mercè Pallàs, Christian Griñán-Ferré

**Affiliations:** 1 Department of Pharmacology and Therapeutic Chemistry. Institut de Neurociències—University of Barcelona, Avda. Joan XXIII, 27. 08028 Barcelona, Spain; vanesa_izquierdo@hotmail.com (V.I.); Vpalomera@hotmail.com (V.P.-Á.); sergio.slr3@gmail.com (S.L.-R.); canudas@ub.edu (A.-M.C.); christian.grinan@ub.edu (C.G.-F.); 2 Department of Cellular and Molecular Biology, University Center of Biological and Agricultural Sciences, University of Guadalajara, km 15.5 Guadalajara-Nogales highway, C.P. 45110 Zapopan, Jalisco, Mexico

**Keywords:** cognitive decline, epigenetics, epigenetic inheritance, methylation, Nrf2, NF-κB, oxidative stress, inflammation, resveratrol, SAMP8

## Abstract

A variety of environmental factors contribute significantly to age-related cognitive decline and memory impairment in Alzheimer’s Disease (AD) and other neurodegenerative diseases. Nutrition can alter epigenetics, improving health outcomes, which can be transmitted across generations; this process is called epigenetic inheritance. We investigate the beneficial effects of maternal resveratrol supplementation in the direct exposed F1 generation and the transgenerational F2 generation. The offspring was generated from females Senescence Accelerated Mouse-Prone (SAMP8) fed a resveratrol-enriched diet for two months prior to mating. Object novel recognition and Morris Water Maze (MWM) demonstrated improvements in cognition in the 6-month-old F1 and F2 generations from resveratrol fed mothers. A significant increase in global DNA methylation with a decrease in hydroxymethylation in F1 and F2 were found. Accordingly, *Dnmt3a/b* and *Tet2* gene expression changed. Methylation levels of *Nrf2* and *NF-kβ* genes promoters raised in offspring, inducing changes in target genes expression, as well as hydrogen peroxide levels. Offspring that resulted from a resveratrol fed mother showed increase AMPKα activation, mTOR inhibition, and an increase in *Pgc-1α* gene expression and Beclin-1 protein levels. Endoplasmic reticulum stress sensors were found changed both in F1 and F2 generations. Overall, our results demonstrated that maternal resveratrol supplementation could prevent cognitive impairment in the SAMP8 mice offspring through epigenetic changes and cell signaling pathways.

## 1. Introduction

Age-related cognitive decline, especially memory impairment, is one of the most prevalent consequences of growing older [1]. The severity of age-related cognitive decline predisposes to neurodegenerative disease such as Alzheimer’s Disease (AD). The molecular mechanisms of age-related cognitive decline and AD are considered multifactorial and implicate the interaction of genetics and environmental factors acting as susceptibility factors or triggers [2] that promote brain injury accumulation due to their chronicity during ageing. Thus, several cell pathways play a significant role in the progression and development of cognitive impairment such as transcriptional dysregulation, Oxidative Stress (OS) [3], inflammation [4], Endoplasmic Reticulum (ER) stress [5], autophagy [6], and retrograde autophagosome transport [7], among others. Recently, an increased understanding of the complexity of age-related cognitive decline has led to define the important role of epigenetics in the pathogenesis [8]. Moreover, mounting evidence indicates that various environmental factors can modulate expression profiles of several genes through epigenetic programs that might modify the susceptibility and variability in the trajectory of age-related cognitive decline and AD [9]. This evidence emphasizes that environmental factors may operate throughout life to influence the biological mechanisms that dictate the cognitive impairment of an individual’s health trajectory.

Until now, it has been accepted that epigenetic changes that occur during life are not generally transmitted into the next generation. This, it has been argued, is because in each generation a developmental reprogramming process that resets the epigenome of the early embryo takes place [10]. Nevertheless, some of the epigenetic alterations have been shown to escape this process, such as global DNA methylation (5-mC), histone modifications, and microRNAs (miRNAs) [11]. Exposure of an organism to different environmental conditions can affect offspring health for the first generation (intergenerational inheritance), or subsequent generations (transgenerational inheritance); this process is called transgenerational epigenetic inheritance [10]. Moreover, several epidemiological studies have shown that the risk of several age-related diseases, including diabetes, cardiovascular disease, as well as cancers and neurodegenerative disorders might largely depend on features of maternal nutrition, stress, toxicants, pathogens, and exercise, among others [11,12]. However, the mechanism underlying this transgenerational memory of environmental factors and its potential consequences across generations remains genuinely unexplored and might involve epigenetic mechanisms. Thus, few studies have attempted to investigate beneficial nutrition outcomes following exposure to a resveratrol maternal diet. Accepting these limitations, recent studies have demonstrated that maternal methyl supplements in mice change 5-mC in offspring [13] and maternal high-fat diet exposure promotes histone modifications in neonatal rats [14]. Furthermore, changes in protein levels of Nrf2 have been reported, a transcription factor that is part of the Antioxidant Response Element (ARE) in the offspring of maternal nutritional intervention [15]. ARE-Nrf2 activates antioxidant enzymes reducing OS, as well as NF-kβ, a transcription factor that actives pro-inflammatory cytokines [16]. Other changes in DNA methylation CpG have been found in various gene promoters [17] and miRNA expression profiles are also found in the offspring after maternal diet intervention in several animal models [18]. 

The Senescence-Accelerated Mouse Prone 8 (SAMP8) is a non-transgenic strain, established through phenotypic selection from the AKR/J strain by Takeda [19]. The SAMP8 strain exhibits age-related cognitive decline, with behavioral abnormalities as well as neuropathological hallmarks of AD [20,21]. Likewise, several key events associated with neurodegeneration are presented in SAMP8 mice such as OS [22,23,24], and inflammatory biomarkers [23]. Recently, epigenetic changes have been associated with accelerated ageing in SAMP8 [25,26], and several studies demonstrated that environmental interventions induced changes in the epigenome, reducing cognitive impairment [25,27].

Resveratrol (3,5,4′-trihydroxy-trans-stilbene) is a phytoalexin found in grapes, peanuts, and wine, with anti-aging, antioxidant, anti-inflammatory, cardioprotective, and antitumor activities [28]. Resveratrol also protects the brain in different neurological conditions, such as AD and Parkinson’s Disease (PD) [29,30,31]. Our group has shown that resveratrol modulates important neuronal functions, improving cognitive decline in several AD mice models [32,33,34]. Although the beneficial effects of resveratrol in the Central Nervous System (CNS) are well established, the mechanisms of these effects are not fully understood but can include epigenetic modulation [35].

The present work aimed to evaluate the neuroprotective effects of maternal resveratrol supplementation in SAMP8 offspring through several key neurodegenerative pathways as well as delving deep into the epigenetic mechanisms influenced by a resveratrol supplementation diet. We focus on OS, neuroinflammation, ER stress, and monophosphate-activated protein kinase (AMPK)/mammalian target of rapamycin (mTOR) cascade signaling. We also evaluated the cognitive performance of the offspring.

## 2. Results

### 2.1. Body Weight Evolution in SAMP8 Offspring after Maternal Resveratrol Supplementation 

**Figure 1:**
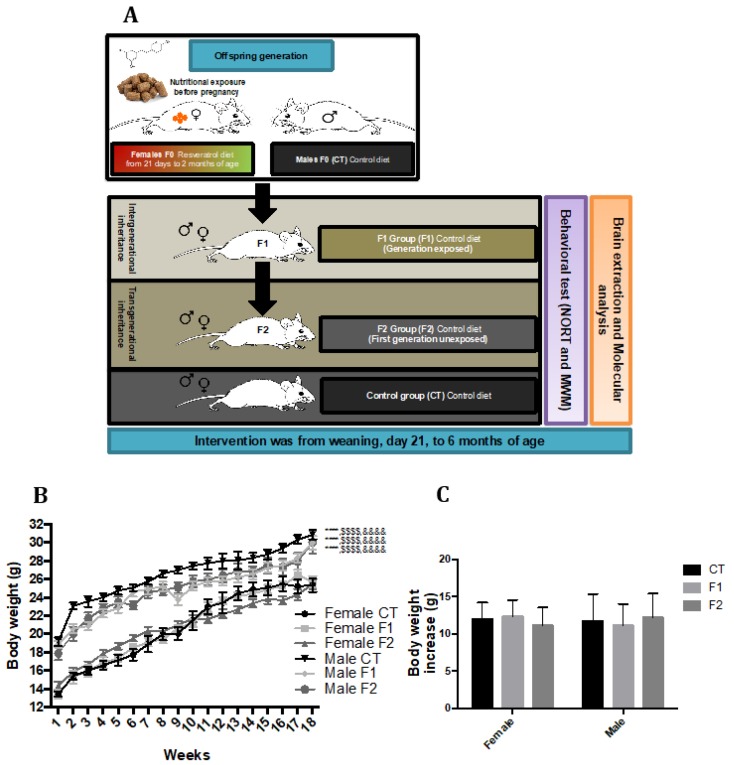
Experimental design and offspring generation of F1 (intergenerational inheritance) and F2 (transgenerational inheritance), and control (CT) mice group. At 6 months of age, mice were tested by memory tests followed by euthanasia, brain extraction, and molecular analysis (**A**). Results of body weight each week in both females and males (**B**). Results of total body weight increase in both females and males (**C**). Values represented are mean ± standard error of the mean (SEM); *n* = 60 (CT *n* = 20, F1 = 20, F2 *n* = 20; for each group, females *n* = 10, males *n* = 10). Statistics: vs. female CT group: **** *p* < 0.0001 vs. female F2 group: ^$$$$^ *p* < 0.0001 vs. female F2 group: ^&&&&^ *p* < 0.0001.

Body weight was measured weekly during the intervention. Starting from baseline (3 weeks of age), all mice groups significantly increased body weight over time, demonstrating the correct development of all groups. Furthermore, no changes in body weight were found within the individual sex groups during the intervention, the only difference in body weight was among male mice groups and female mice groups (Figure 1B). The average body weight increase was 11.953 g for CT group, 12.305 g for F1 group, and 11.130 g for F2 group from week three to week 17 in female mice groups. Likewise, the average body weight increase was 11.713 g for CT group, 11.109 g for F1 group, and 12.162 g for F2 group from week three to week 17 in female mice groups (Figure 1C). 

### 2.2. Maternal Resveratrol Supplementation Mitigate Cognitive Impairment Presented by SAMP8 Across Generations 

**Figure 2:**
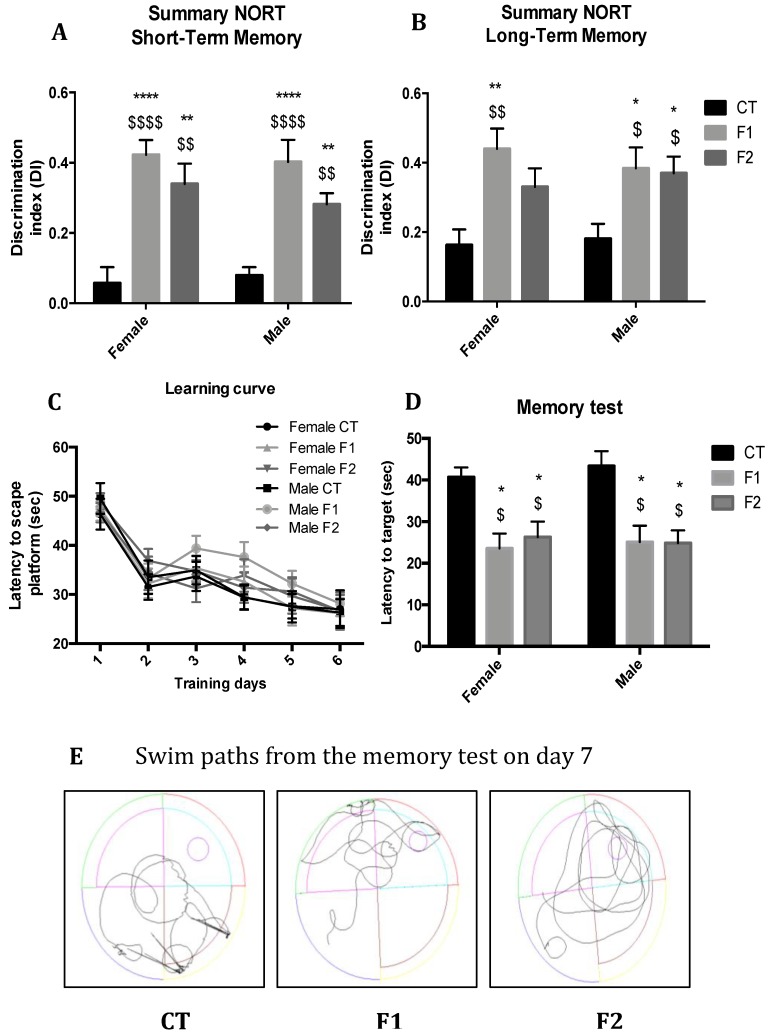
Results of DI of Novel Object Recognition Test (NORT) in females and males at 6 months of age in both females and males for all mice groups. Summary from short-term memory (**A**), and a summary from long-term memory (**B**). Results of Morris Water Maze (MWM) in females and males at 6 months of age for all mice groups. Learning curves of MWM during the spatial acquisition phase (**C**). Latency to the target of MWM on the memory test (**D**). Representative swim paths on the memory test (**E**). Values represented are mean ± SEM; *n* = 60 (CT *n* = 20, F1 = 20, F2 *n* = 20; for each group, females *n* = 10, males *n* = 10). Statistics: vs. female CT group: * *p* < 0.05; ** *p* < 0.01; **** *p* < 0.0001 vs. male CT group: ^$^ *p* < 0.05; ^$$^ *p* < 0.01; ^$$$$^ *p* < 0.0001.

In the Novel Object Recognition Test (NORT) analysis we demonstrated that F1 and F2 mice groups exhibited a significant improvement in cognitive performance in both short- and long-term memory in comparison with the CT mice group of both sexes (Figure 2A,B), showing higher Discrimination Index (DI) with statistically significant differences in both females and males, but not among gender. Furthermore, in the Morris Water Maze (MWM) test, all mice groups were able to learn through training days; no differences were found comparing groups (Figure 2C). On the test day, it was observed that the CT mice group showed deficits in spatial memory, with a significant increase in the latency to the target and erratic swim path in comparison with F1 and F2 mice groups in both sexes (Figure 2D,E). As in NORT, no gender differences were found. 

### 2.3. Global Changes in DNA Methylation and Hydroxymethylation and its Epigenetic Modulators in the Hippocampus of SAMP8 Offspring 

**Figure 3:**
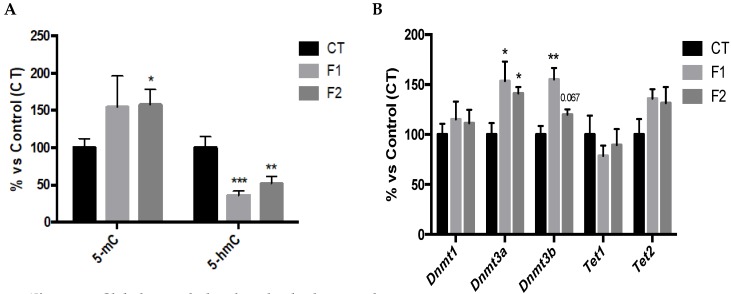
Global 5-methylated and 5-hydroxymethylated cytosine levels in the hippocampus of females and males at 6 months of age for all mice groups (**A**). Relative gene expression of *Dnmt1*, *Dnmt3a, Dnmt3b, Tet1*, and *Tet2* (**B**). Gene expression levels were measured by real-time polymerase chain reaction (PCR) from hippocampal mRNA. Mean ± SEM in bar graphs are adjusted to 100% for each gene of CT group; *n* = 18 to 21 (CT *n* = 6 to 7, F1 *n* = 6 to 7, F2 *n* = 6 to 7; for each group, females 3 to 4, males *n* = 3). Statistics: * *p* < 0.05; ** *p* < 0.01; *** *p* < 0.001.

5-mC levels were increased in F1 and significantly increased in F2 in comparison with the CT mice group (Figure 3A). Likewise, 5-hmC levels were significantly reduced in both F1 and F2 in comparison with the CT mice group (Figure 3A). In parallel, DNA Methyltransferases family (DNMTs) gene expression was evaluated and results demonstrated a significant increase in *Dnmt3a* and *Dnmt3b* in both F1 and F2 mice groups (Figure 3B). Ten-Eleven Translocation family (TETs) gene expression was also evaluated, and no changes were found in *Tet1* as well as slightly increased in *Tet2* in both F1 and F2 mice groups compared to the CT group (Figure 3B). No gender differences were found, and, for this reason, results are presented merging both genders.

### 2.4. Changes in Methylation levels of Nrf2 and NF-kβ Gene Promoters and Gene Expression of their Targets reduce Oxidative Stress and Inflammation in the Hippocampus of SAMP8 Offspring

**Figure 4:**
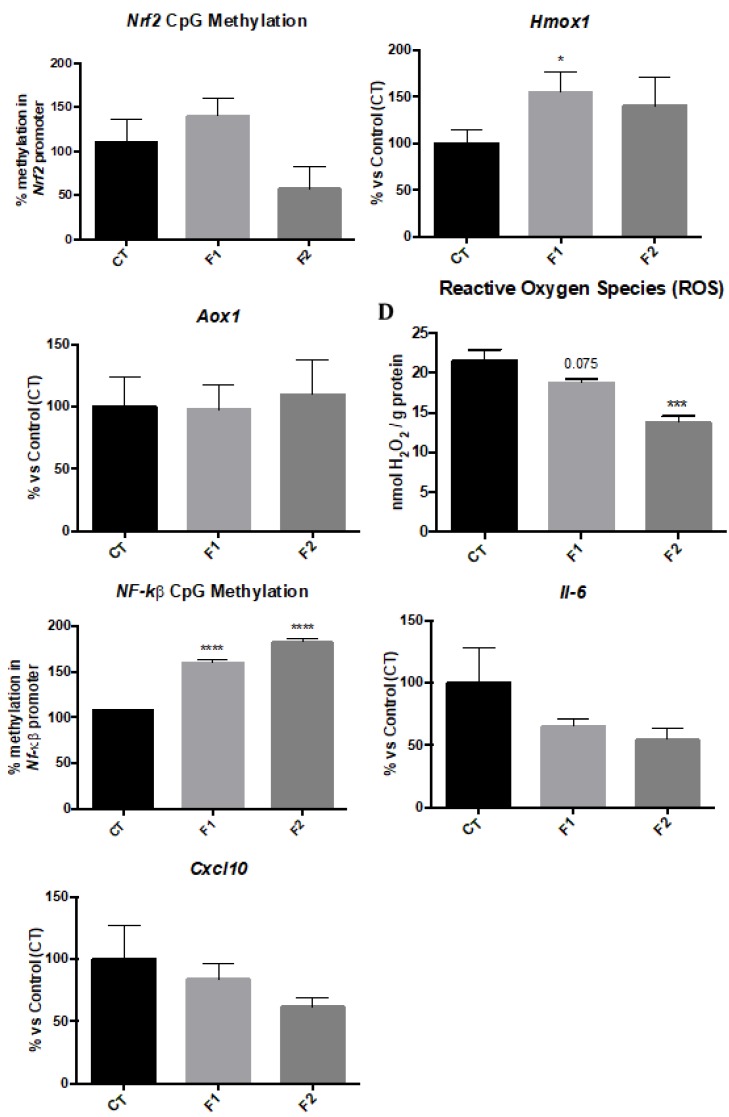
DNA methylation analysis of the *Nrf2* (**A**) and *NF-kβ* (**E**) gene promoter in the hippocampus of females and males at 6 months of age for all mice groups. Percentage of gene methylation was determined by real-time PCR. Mean ± values in bar graphs are adjusted to 100% for each gene of the CT mice group. Representative Oxidative stress measured as hydrogen peroxide concentration in homogenates of hippocampus tissue (**D**). Representative gene expression for *Hmox1* (**B**), *Aox1* (**C**), *Il-6* (**F**), *Cxcl10* (**G**). Gene expression levels were measured by real-time PCR from hippocampal mRNA. Mean ± SEM in bar graphs are adjusted to 100% for each gene of the CT; *n* = 18 (CT *n* = 6, F1 *n* = 6, F2 *n* = 6; for each group, females *n* = 3, males *n* = 3). Statistics: * *p* < 0.05; *** *p* < 0.001.

We studied the promoter methylation levels of transcription factor genes *Nrf2* and *NF-kβ*. The *Nrf2* promoter methylation levels showed a reduction only in the F2 mice group in comparison with the CT group (Figure 4A). In parallel fashion, gene expression of Heme oxygenase (decycling) 1 (*Hmox1*), a target gene of *Nrf2* was upregulated in both F1 and F2 mice groups, being only significant in F1 compared to the CT group (Figure 4B). Finally, the analysis of hydrogen peroxide levels in homogenates of the hippocampus showed a reduction in Radical Oxygen Species (ROS) levels in F1 and a significant reduction in F2 in comparison with the CT group mice (Figure 4D). Furthermore, the promoter methylation of *NF-kβ* gene was studied, and a significant increase in both F1 and F2 mice groups were found in comparison with the CT group (Figure 4E). Likewise, we found that this increase in promoter methylation levels rendered a reduction in *Il-6* and *Cxcl10* gene expression in both F1 and F2 mice groups in comparison with the CT mice group (Figure 4F,G). No gender differences were found, and, for this reason, results are presented merging both genders.

### 2.5. Changes in AMPK/mTOR Signalling Cascade in the Hippocampus of SAMP8 Offspring 

**Figure 5:**
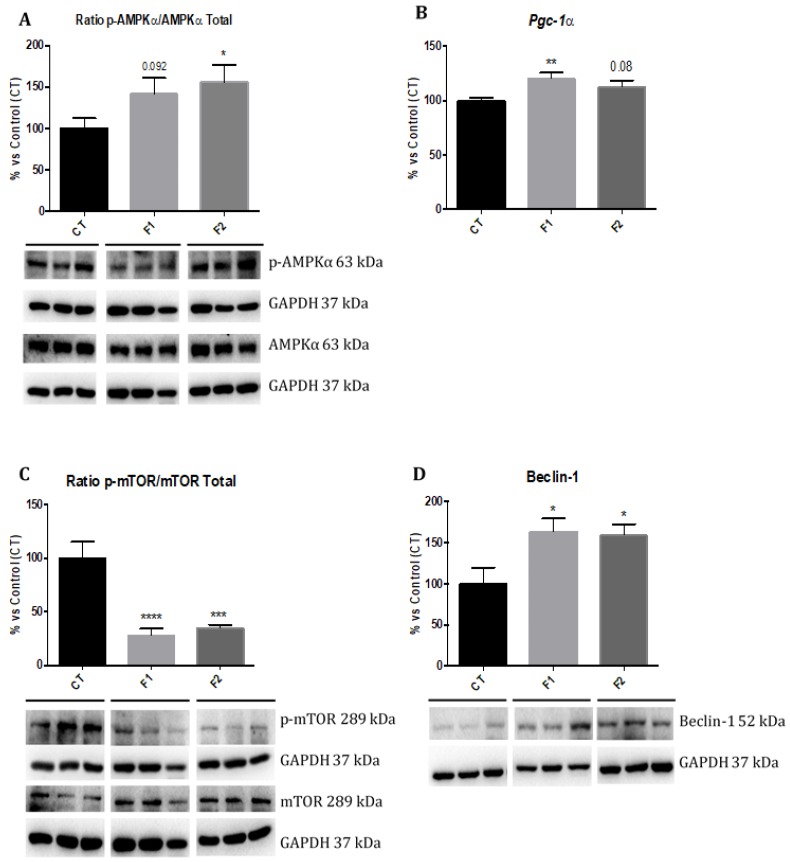
AMPK/mTOR signaling cascade in the hippocampus of females and males at 6 months of age for all mice groups. Representative Western blot for ratio p-AMPK/AMPK, ratio p-mTOR/mTOR, Total and Beclin-1 protein levels, and their respective quantification (**A**,**C**,**D**). Values in bar graphs are adjusted to 100% for protein levels of the CT group. Representative gene expression for *Pgc-1α* (**B**). Gene expression levels were measured by real-time PCR from hippocampal mRNA. Values are mean ± SEM; *n* = 18 (CT *n* = 6, F1 *n* = 6, F2 *n* = 6 for each group, females *n* = 3, males *n* = 3). Statistics: *** *p* < 0.001; **** *p* < 0.0001.

Western blot analysis revealed a significant increase in phosphorylated AMPK (p-AMPK) levels in F2, but no changes were found in the F1 mice group in comparison with the CT group (Figure 5A). Moreover, a significant increase in gene expression of *Pgc-1α* in F1 as well as a clear tendency to increase in F2 mice group, compared to the CT group (Figure 5B). In parallel, a significant reduction in phosphorylated mTOR (p-mTOR) levels was found in both F1 and F2 in comparison with the CT group (Figure 5C). Finally, a significant increase in Beclin-1 protein levels in both F1 and F2 mice groups in comparison with the CT mice group were found (Figure 5D). No gender differences were found, and, for this reason, results are presented merging both genders.

### 2.6. Reduction in ER Stress in the Hippocampus of SAMP8 Offspring

**Figure 6:**
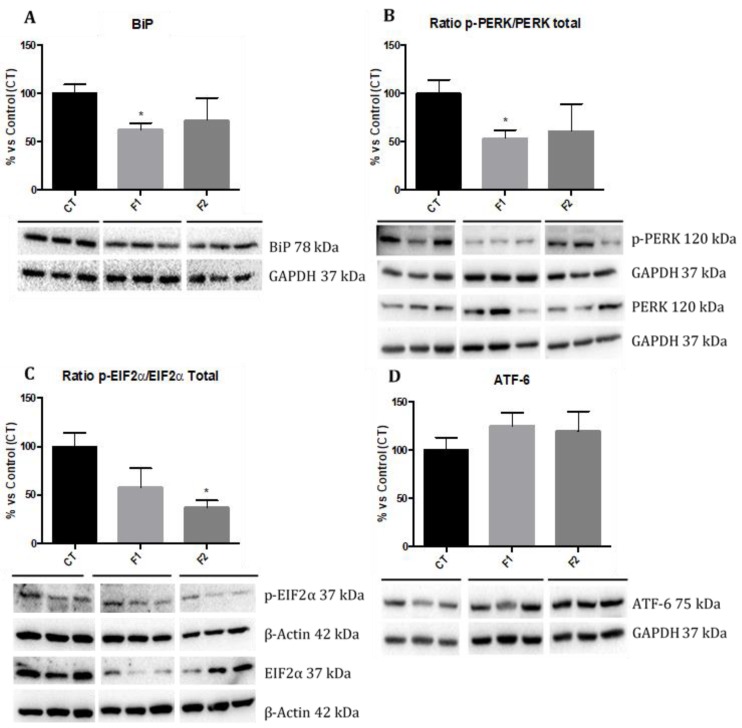
Endoplasmic Reticulum (ER) stress markers sensors in the hippocampus of females and males at 6 months of age for all mice groups. Representative Western blot for BiP), ratio p-PERK/PERK Total, ratio p-EIF2α/EIF2α, ATF-6 and their respective quantification (**A**–**D**). Values in bar graphs are adjusted to 100% for protein levels of the CT group). Values are the mean ± SEM; *n* = 18 (CT *n* = 6, F1 *n* = 6, F2 *n* = 6 for each group, females *n* = 3, males *n* = 3). Statistics: * *p* < 0.05.

We evaluated the ER stress markers such as Immunoglobulin binding Protein (BiP), phosphorylated protein kinase R-line Endoplasmic Reticulum Kinase (p-PERK), phosphorylated Eukaryotic Translation-initiation Factor 2 (p-EIF2α), and Activating Transcription Factor 6 (ATF-6) by Western blot. Analysis revealed that protein levels of BiP and p-PERK were significantly reduced only in the F1 group, although a reduction was also found in the F2 group in comparison with the CT group mice (Figure 6A,B). Regarding p-EIF2α levels, a significant reduction in the F2 group and slightly decreased in the F1 mice group were observed in comparison with the CT group (Figure 6C). Finally, no changes in ATF-6 protein levels were found among mice groups (Figure 6D). No gender differences were found, and, for this reason, results are presented merging both genders.

## 3. Discussion

The present study investigated, the beneficial effect of maternal resveratrol supplementation 2 months before conception on offspring over two generations in the SAMP8 mouse model. Thus, cognitive performance, several molecular pathways influenced by an age-related cognitive decline as well as 5-mC and 5-hmC and the machinery involved in these marks and DNA methylation levels of CpG gene promoters were studied to elucidate the resveratrol-induced epigenetic transgenerational inheritance mechanism. The intervention was finished at the age of 6-months-old when cognitive impairment and main molecular mechanisms of neurodegeneration such as OS, inflammation, among others are well characterized in female and male of SAMP8 [36,37]. 

We have previously described that resveratrol dietary supplementation induced changes in cognition and molecular pathways in the hippocampus of aged [38,39] and AD mice models [33,40]. Here, we reported that body weight remained unchanged in the offspring from females supplemented with resveratrol before pregnancy in comparison with the sex control group, confirming the same effect in both sexes elicited by polyphenol, and corroborating previous studies [41,42]. Furthermore, we demonstrated that in two generations of progenies from females exposed to resveratrol supplementation before pregnancy displayed improvement in recognition (NORT) and spatial memories (MWM) in both sexes. Thus, no sex-biased multigenerational effect in the offspring outcomes induced by maternal resveratrol prior to mating was found. Consistent with our new findings, it has been reported that the neuroprotective effects of resveratrol, against cognitive impairment induced by prenatal stress in the offspring of Wistar rats [43].

As mentioned above, an increasing number of studies in rodents have demonstrated that the nutritional stimuli may alter the phenotype of animals through epigenetic mechanisms [44] as well as these epigenetic alterations being transmitted through at least three generations in mammals [45]. Here, we found a higher degree of global 5-mC and a diminution in 5-hmC, which paralleled with changes in their enzymatic machinery such as DNMTs and TETs, in the hippocampus of the offspring. These epigenetic marks have been described as essential for hippocampal synaptic plasticity, cognitive function [46], and age-related modifications [47]. In line with our results, several studies found that dietary polyphenols alter epigenetic mechanisms by changing 5-mC levels [48] or regulating the gene expression or enzymatic activity of DNMTs and TETs [49]. In addition, recent studies suggest that maternal resveratrol intake after different stimuli can have a significant influence on the susceptibility of the offspring to age-related diseases including cognitive decline [44,50,51]. Nevertheless, to our knowledge, this is the first study in which only a maternal resveratrol supplementation before pregnancy has been shown to lead to beneficial effects on cognition in the offspring of SAMP8, being the action orchestrated through epigenetic mechanisms. 

Because of the improvement in cognitive function observed in the offspring, it was of interest to determine which of the cellular mechanisms are implicated in the neuroprotective effects of resveratrol-mediated by epigenetic changes demonstrated. In this way, OS and inflammation pathways can be regulated through epigenetic mechanisms, hence we first studied the methylation status of two transcriptional regulatory gene promoters; Nrf2 a transcription factor that plays a crucial role in protection against ROS, binds to the ARE, and regulates antioxidant enzymes [52] and NF-kβ, another transcription factor that regulates a large number of genes involved in the inflammatory response [53]. Gene transcription is activated when specific CpG sites are demethylated and, conversely, silenced when sites are methylated [54]. Here, we found that methylation levels in the promoter of the Nrf2 gene were lower in the F2 offspring. *Hmox1* gene expression, but not Aldehyde oxidase 1 (*Aox1*), was increased in both generations. HMOX1 protein can protect against OS [55], whereas AOX1 mediated ROS [27]. Moreover, higher methylation levels in the promoter of the NF-kβ in both generations were found, confirming the lower gene expression levels of *Il-6* and *Cxcl10.* Therefore, these results demonstrated the reduction in ROS and pro-inflammatory cytokines in the hippocampus of the two generations from females exposed to resveratrol. These findings are consistent with reports in other maternal resveratrol intake interventions in which the offspring presented a decrease in OS levels in nonhuman primates [56], and reduced secretion and expression of pro-inflammatory markers in rodents [57] and humans [51]. It is also important that cognitive improvement in Nrf2- and NF-kβ levels modulation have been described in SAMP8 after environmental enrichment (EE) [27] and a rat kidney ischemia model [58], among others.

In addition to antioxidant and anti-inflammatory effects, it is well established that resveratrol has a number of indirect effects [59,60], including mitochondrial biogenesis by AMPK activation [61] and autophagy by inhibition of mTOR [62]. Accordingly, our results showed that the neuroprotective effects of resveratrol in the offspring could be explained, in part, by the activation of the AMPK/mTOR signaling pathway. For instance, higher levels of p-AMPK/AMPK ratio with a concomitant increase of *Pgc-1α* gene expression in the offspring from females supplemented with resveratrol compared to the control group were observed, being practically significant in both generations. Furthermore, a drastic decline of p-mTOR/mTOR ratio and significantly increased protein levels of Beclin-1 were found. Although similar results were found in resveratrol-treated animals and the offspring after maternal resveratrol exposure [51,63], here it is shown that the beneficial effects of resveratrol were maintained unexpectedly across generations. It is of paramount importance demonstrate that molecular mechanisms implicated in the intergenerational and transgenerational resveratrol effects occur through the contact with polyphenol, reinforcing the importance of epigenetic machinery in the inheritance across generations.

Finally, our study revealed that offspring from females supplemented with resveratrol resulted in amelioration of ER stress marker proteins as emphasized by a drastic reduction of p-PERK/PERK ratio and p-EIF2α/EIF2α ratio in the hippocampus. Likewise, a reduction in BiP and no changes in ATF-6 protein levels were found in the offspring. This finding concurs with the report, which demonstrated that pre-treatment with resveratrol attenuated cognitive impairment and decreased ER stress markers in aged mice [64], as well as the modulatory effects of resveratrol on ER stress in a rat model of PD, restoring locomotor activity [65], through the suppression of ER stress via PERK/EIF2α pathway by resveratrol.

**Figure 7:**
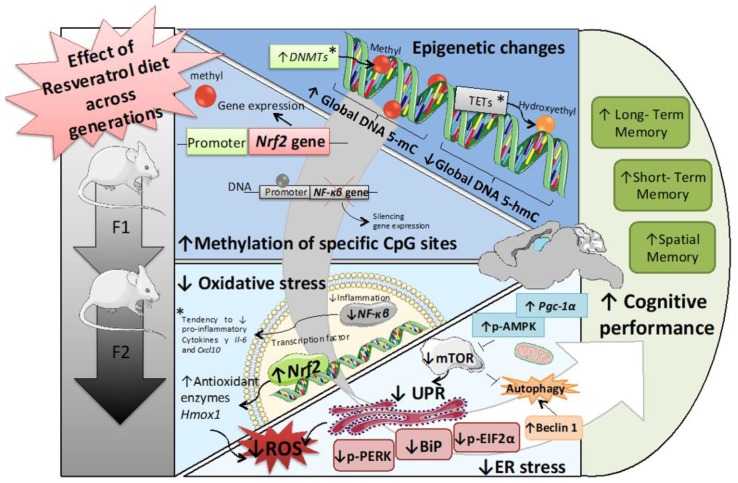
Representative scheme of epigenetic and molecular mechanisms modified in the hippocampus of Senescence-Accelerated Prone Mice (SAMP8) across generations. Significant modifications are shown in bold and potential modifications with the symbol *.

Taken together, our study provides new evidence that maternal resveratrol supplementation can delay the progression of several signs of neurodegeneration, such as cognitive impairment in the next two generations. Interestingly, those effects appeared both in male and female offspring. These results demonstrate that resveratrol acts by modifying epigenetic marks and their machinery as well as methylation levels of CpG in *Nrf2* and *NF-kβ* gene promoters, thus reducing ROS levels and inflammation. Besides, changes in the AMPK/mTOR signaling cascade and ER stress sensor markers explain the beneficial effects induced by resveratrol in the hippocampus of SAMP8 offspring and confirm that maternal dietary interventions before pregnancy can be a promising experimental strategy to elucidate the underlying mechanisms of age-related cognitive decline (Figure 7).

## 4. Material and Methods

### 4.1. Animals

Offspring generation was carried out from two diet groups of SAMP8 in which mice were fed either a standard chow diet (Males F0 (CT), *n* = 2) that had access to standard chow and a resveratrol group (Females F0 (Resveratrol, *n* = 4) that had access to diet enriched with the resveratrol (1 g/Kg) for two months. We interrupted the supplementation diet for mating to take place, obtaining the first generation (the intergenerational inheritance offspring (F1, *n* = 20; females *n* = 10, males *n* = 10)). Afterwards, we crossed the F1 and obtained the second generation (the transgenerational inheritance offspring (F2, *n* = 20; females *n* = 10, males = 10)) (Figure 1A). These two generations were fed a standard diet. This procedure was carried out to advance one generation to obtain the transgenerational inheritance in the F2 generation instead of the F3 generation. On the other hand, we generated the SAMP8 control group (CT, *n* = 20; females *n* = 10, males *n* = 10) that had access to standard chow. Animals had free access to food and water and were kept under standard temperature conditions (22 ± 2 °C) and 12 h: 12 h light-dark cycles (300 lux/0 lux). 

All experimental procedures involving animals were performed followed by standard ethical guidelines European Communities Council Directive 86/609/EEC and by the Institutional Animal Care and Use Committee of the University of Barcelona (670/14/8102, approved at 11/14/2014) and by Generalitat de Catalunya (10291, approved 1/28/2018). All efforts were made to minimize the number of mice used and their suffering.

### 4.2. Behavioral Tests

#### 4.2.1. Novel Object Recognition Test

The NORT protocol employed was a modification of [66,67]. In brief, mice were placed in a 90°, two-arms, 25-cm-long, 20-cm-high, 5-cm-wide black maze. Before performing the test, the mice were individually habituated to the apparatus for 10 min for 3 days. On day 4, the animals were submitted to a 10-min acquisition trial (first trial), during which they were placed in the maze in the presence of two identical, novel objects at the end of each arm. After a delay (2 h and 24 h), the animal was exposed to two objects one old object and one novel object. The time that mice explored the Novel object (TN) and Time that mice explored the Old object (TO) were measured. A DI was defined as (TN − TO)/(TN + TO). To avoid object preference biases, objects were counterbalanced.

#### 4.2.2. Morris Water Maze Test

An open circular pool (100 cm in diameter, 50 cm in height) was filled halfway with water [68], and the temperature was maintained at 22 °C ± 1. Two principal perpendicular axes were defined; thus, the water surface was divided into four quadrants (NE, SE, SW, and NW) and five starting points were set (NE, E, SE, S, and SW). Four visual clues were placed on the walls of the tank (N, E, S, and W). Non-toxic, white latex paint was added to make the water opaque, and a white escape platform was submerged 1.5 cm below the water level (approximately in the middle of one of the quadrants).

The animals swimming paths were recorded by a video camera mounted above the center of the pool, and data were analyzed with SMART version 3.0 software. The acquisition phase consisted of 6 days of trials for each mouse. The animals were submitted to five trials each day starting from the positions set (in random order) and without a resting phase between each trial and the subsequent one. At each trial, the mouse was placed gently into the water, facing the wall of the pool, and allowed to swim for 60 s. If not able to locate the platform in this period, the mouse was guided to the platform by the investigator. Animals were left on the platform each time for 20 s to allow for spatial orientation. A memory test was performed at the end of the training days, in which the platform was removed and the latency to the target, among other parameters by each mouse, was measured.

### 4.3. Immunodetection Experiments

#### 4.3.1. Brain Processing and Protein Determination

Mice were euthanized one day after the behavioral test finished by cervical dislocation. Brains were immediately removed from the skull. The hippocampus was then isolated and frozen on powdered dry ice. They were maintained at −80 °C for further use. Tissue samples were homogenized in lysis buffer (50 mM Tris-HCl, 150 mM NaCl, 5 mM EDTA, 1% Triton X-100, pH 7.4), EDTA-free Protease inhibitor cocktail (Roche, Mannheim, Germany) and Phosphatase inhibitor cocktail II (Sigma-Aldrich, St. Louis, MO, USA). Total proteins were extracted and quantified by the Bradford method. 

#### 4.3.2. Western Blotting 

For Western Blotting, aliquots of 15 µg of hippocampal protein were used. Protein samples were separated by Sodium Dodecyl Sulfate-PolyAcrylamide Gel Electrolysis (SDS–PAGE) (8% to 18%) and transferred into Polyvinylidene difluoride (PVDF) membranes (Millipore). The membranes were blocked in 5% non-fat milk in TBS containing 0.1% Tween 20 (TBS-T) for 1 h at room temperature, followed by overnight incubation at 4 °C with the primary antibodies listed in Table S1. Membranes were then washed and incubated with secondary antibodies for 1 h at room temperature. Immunoreactive protein was viewed with a chemiluminescence-based detection kit, following the manufacturer’s protocol (ECL Kit; Millipore, Billerica, MA, USA), and digital images were acquired using a ChemiDoc XRS + System (BioRad, Hercules, CA, USA). Semi-quantitative analyses were carried out using Image Lab software (BioRad), and results were expressed in Arbitrary Units (AU) considering control protein levels as 100%. Protein loading was routinely monitored by immunodetection of Glyceraldehyde-3-phosphate dehydrogenase (GADPH) or β-actin.

#### 4.3.3. RNA Extraction and Gene Expression Determination

Total RNA isolation was carried out using TRIzol^®^ reagent according to the manufacturer’s instructions (Invitrogen, Carlsbad, CA, USA). The yield, purity, and quality of RNA were determined spectrophotometrically with a NanoDrop™ ND-1000 (Thermo Scientific, Wilmington, DE, USA apparatus and an Agilent 2100B Bioanalyzer (Agilent Technologies, Palo Alto, CA, USA). RNAs with 260/280 ratios and RIN higher than 1.9 and 7.5, respectively, were selected. Reverse Transcription-Polymerase Chain Reaction (RT-PCR) was performed as follows: 2 μg of messenger RNA (mRNA) was reverse-transcribed using the High Capacity cDNA Reverse Transcription Kit (Applied Biosystems, Foster City, CA, USA. Real-time quantitative PCR (qPCR) was used to quantify mRNA expression of chromatin-modifying genes as well as to validate selected genes from microarray data results. 

SYBR^®^ Green real-time PCR was performed in a Step One Plus Detection System (Applied-Biosystems) employing SYBR^®^ Green PCR Master Mix (Applied-Biosystems). Each reaction mixture contained 6.75 μL of complementary DNA (cDNA) (which concentration was 2 μg/μL), 0.75 μL of each primer (which concentration was 100 nM), and 6.75 μL of SYBR^®^ Green PCR Master Mix (2×). 

TaqMan-based real-time PCR (Applied Biosystems) was also performed in a Step One Plus Detection System (Applied-Biosystems). Each 20 μL of TaqMan reaction contained 9 μL of cDNA (25 ng), 1 μL 20× probe of TaqMan Gene Expression Assays and 10 μL of 2× TaqMan Universal PCR Master Mix. 

Data were analyzed utilizing the comparative Cycle threshold (C*t*) method (ΔΔC*t*), where the housekeeping gene level was used to normalize differences in sample loading and preparation. Normalization of expression levels was performed with *β-actin* for SYBR^®^ Green-based real-time PCR results and *Gapdh* for TaqMan-based real-time PCR. Primers and TaqMan probes are listed in Table S2. Each sample was analyzed in duplicate, and the results represent the n-fold difference of the transcript levels among different groups.

#### 4.3.4. Global DNA Methylation and Hydroxymethylation Determination

Isolation of genomic DNA was conducted using the FitAmp^TM^ Blood and Cultured Cell DNA Extraction Kit (EpiGentek, Farmingdale, NY, USA) according to the manufacturer’s instructions. Following this, Methylflash Methylated DNA Quantification Kit (Epigentek, Farmingdale, NY, USA) and MethylFlash HydroxyMethylated DNA Quantification Kit were used in order to detect methylated and hydroxymethylated DNA. Briefly, these kits are based on specific antibody detection of 5-mC and 5-hmC residues, which trigger an ELISA-like reaction that allows colorimetric quantification by reading absorbance at 450 nm using a Microplate Photometer. The absolute amount of methylated or hydroxymethylated DNA (proportional to the Optical Density (OD) intensity) was measured and quantified using a standard curve plotting OD values vs. five serial dilutions of a control methylated and hydroxymethylated DNA (0.5 to 10 ng). 

#### 4.3.5. Genomic DNA Extraction, Bisulfite DNA Conversion and Methylation-specific PCR (MSP)

Genomic DNA was isolated from the hippocampus using the FitAmp^TM^ Blood and Cultured DNA Extraction Kit (EpiGentek, Farmingdale, NY, USA) according to the manufacturer’s instructions. DNA concentration and purity were quantified using NanoDrop™ ND-1000 (Thermo Scientific). This was followed by bisulfite conversion being performed using the BisulFlash DNA Modification Kit from Epigentek (Farmingdale, NY, USA). Following this, the unmethylated cytosine residues were modified into uracil, while the methylated cytosine residues were preserved. As a result, the sodium bisulfite-treated DNA of the methylated and the unmethylated differed and was distinguishable by methyl-specific PCR primers designed to have complementary sequences to the formerly unmethylated and methylated genome. The primer sequences can be found in Table S3. DNA amplification was performed using reagents prepared by Methylamp MS-PCR Fast Kit from Epigentek (Farmingdale, NY, USA). The thermal profile consisted of 95 °C for 7 min as the initial denaturation step, 40 cycles of 95 °C for 10 s, 55 °C for 10 s, 72 °C for 60 s for DNA amplification, followed by a final extension step of 72 °C for 1 min. PCR reactions were performed in 96-well plates in a Step One Plus Detection System (Applied Biosystems).

#### 4.3.6. Determination of Oxidative Stress in Hippocampus

Hydrogen peroxide was measured as an indicator of oxidative stress and it was quantified using the Hydrogen Peroxide Assay Kit (Sigma-Aldrich, St. Louis, MI, USA) according to the manufacturer’s instructions.

#### 4.3.7. Data Analysis

The statistical analysis was conducted using GraphPad Prism ver. 6 statistical software. Data are expressed as the mean ±SEM of at least 4 samples per group. Sex and group effects for body weight and behavioral tests were assessed by the Two-Way Analysis of variance, followed by Tukey post-hoc analysis. Molecular analysis was assessed by The One-Way Analysis of variance (ANOVA), followed by Tukey post-hoc analysis or two-tail Student’s t-test when it was necessary. Statistical significance was considered when *p*-values were <0.05. The statistical outliers were determined with Grubbs’ test and subsequently removed from the analysis.

## Supplementary Materials

Supplementary materials can be found at https://www.mdpi.com/1422-0067/20/5/1134/s1. **Table S1.** Antibodies used in Western blot studies. **Table S2.** Primers and probes used in qPCR studies. **Table S3.** Primer sequences for the MSP primers.
