# Supplementary material for: Maternal Resveratrol Supplementation Prevents Cognitive Decline in Senescent Mice Offspring
Source: Int J Mol Sci. 2019 Mar 6;20(5):1134. doi: 10.3390/ijms20051134 (PMC6429303; doi:10.3390/ijms20051134)
Supplement: Supplementary file 1 [file ijms-20-01134-s001.pdf]

Table S1. Antibodies used in Western blot studies.

Antibody	Host	Source/Catalog	WB dilution
**AMPK**	Rabbit	Cell Signaling/#2532	1:1000
**p-AMPK**	Rabbit	Cell Signaling/#2537	1:1000
**mTOR**	Rabbit	Millipore/MABS14	1:1000
**p-mTOR**	Rabbit	Millipore/09-213	1:1000
**PERK**	Rabbit	Cell Signaling/#3192	1:1000
**p-PERK**	Rabbit	Cell Signaling/#3179	1:1000
**ATF-6**	Rabbit	Cell Signaling/#65880	1:1000
**BiP**	Rabbit	Cell Signaling/D14E12	1:1000
**EIF2α**	Rabbit	Cell Signaling/#9722	1:1000
**p-EIF2α**	Rabbit	Cell Signaling/#9721	1:1000
**β -Actin**	Mouse	Sigma/A-5441	1:10000
**GAPDH**	Mouse	Millipore/MAB374	1:5000
**Goat-anti-mouse HRP conjugated**		Biorad/#170-5047	1:2000
**Goat-anti-rabbit HRP conjugated**		Cell Signaling/#7074	1:2000

Table S2. Primers and probes used in qPCR studies.

SYBR Green primers

Target	Product size (bp)	Forward primer (5'-3')	Reverse primer (5'-3')
Tet2	113	CCATCATGTTGTGGGACGGA	ATTCTGAGAACAGCGACGGT
Il-6	189	ATCCAGTTGCCTTCTTGGGA CTGA	TAAGCCTCCGACTTGTGAAG TGGT
Cxcl10	72	GGCTAGTCCTAATTGCCCTT GG	TTGTCTCAGGACCATGGCTT G
Hmox1	177	TGACACCTGAGGTCAAGCAC	GTCTCTGCAGGGGCAGTATC
Aox1	286	CATAGGCGGCCAGGAACATT	TCCTCGTTCCAGAATGCAGC
Pgc-1α	274	GGCCTAACTCCTCCCACAAC	GGTCACCAAACAGCCGAAG A
β-Actin	190	CAACGAGCGGTTCCGAT	GCCACAGGTTCCATACCCA

Taqman probes

Target	Product size (bp)	Reference
Dnmt1	58	Mm01151063_m1
Dnmt3a	58	Mm00432881_m1
Dnmt3b	83	Mm01240113_m1
Tet1	69	Mm01169087_m1
Gapdh	107	Mm99999915_g1

Table 3S. Primer sequences for the MSP primers.

mRNA Primer (5'-3')	Denaturing ºC (s)	Annealing ºC (s)	Elongation ºC (s)	Cycles
Nrf2	95 (10)	55 (10)	72 (60)	40
**F: TTCGTTGTTCGGATTAGTTA**<br>**R: CCGACGACGAAACTACTTA**				
Nf-kβ	95 (10)	55 (10)	72 (60)	40
**F: AAGAGTTTCGAGACGTTATTC**<br>**R: CTTCTCCTCCCGCTAACTA**				
